# Advanced Artificial Intelligence Technologies Transforming Contemporary Pharmaceutical Research

**DOI:** 10.3390/bioengineering12040363

**Published:** 2025-03-31

**Authors:** Parveen Kumar, Benu Chaudhary, Preeti Arya, Rupali Chauhan, Sushma Devi, Punit B. Parejiya, Madan Mohan Gupta

**Affiliations:** 1 Department of Pharmaceutics, NIMS Institute of Pharmacy, NIMS University, Jaipur 303121, Rajasthan, India; praveenmoond@gmail.com; 2 Shri Ram College of Pharmacy, Karnal 132001, Haryana, India; benuchaudhary2@gmail.com (B.C.); aryapreeti501@gmail.com (P.A.); 3 Chitkara College of Pharmacy, Chitkara University, Rajpura 140401, Punjab, India; rupali18chauhan@gmail.com (R.C.); sushma.mehla@gmail.com (S.D.); 4 Department of Pharmaceutics, K.B. Institute of Pharmaceutical Education and Research, Kadi Sarva Vishwavidyalaya, Gandhinagar 382 023, Gujarat, India; punit.parejiya@kbiper.ac.in

**Keywords:** artificial intelligence, computational learning, healthcare, drug discovery, personalized medicine, patient, structural activity relationship

## Abstract

One area of study within machine learning and artificial intelligence (AI) seeks to create computer programs with intelligence that can mimic human focal processes in order to produce results. This technique includes data collection, effective data usage system development, conclusion illustration, and arrangements. Analysis algorithms that are learning to mimic human cognitive activities are the most widespread application of AI. Artificial intelligence (AI) studies have proliferated, and the field is quickly beginning to understand its potential impact on medical services and investigation. This review delves deeper into the pros and cons of AI across the healthcare and pharmaceutical research industries. Research and review articles published throughout the last few years were selected from PubMed, Google Scholar, and Science Direct, using search terms like ‘artificial intelligence’, ‘drug discovery’, ‘pharmacy research’, ‘clinical trial’, etc. This article provides a comprehensive overview of how artificial intelligence (AI) is being used to diagnose diseases, treat patients digitally, find new drugs, and predict when outbreaks or pandemics may occur. In artificial intelligence, neural networks and deep learning are some of the most popular tools; in clinical research, Bayesian non-parametric approaches hold promise for better results, while smartphones and the processing of natural languages are employed in recognizing patients and trial monitoring. Seasonal flu, Ebola, Zika, COVID-19, tuberculosis, and outbreak predictions were made using deep computation and artificial intelligence. The academic world is hopeful that AI development will lead to more efficient and less expensive medical and pharmaceutical investigations and better public services.

## 1. Introduction

The study of how computers can learn to solve problems using symbolic language has been termed as artificial intelligence (AI). Many fields, including business, medicine, technology, and more, have benefited from its development, and it has become a core research method for resolving issues [1]. Finding practical issues with interpreting data and providing a vague outline of how to fix them is the primary goal of this type of AI. This goal is known to be a direct approach, and it lines up with extant mathematical theorem. The study and practice of developing and implementing programs to feed the purpose of analyzing information, learning, and decoding is known as artificial intelligence (AI). There are many different aspects that fall under the umbrella of artificial intelligence, including analytical and computational learning, identifying patterns, classification, and other techniques [2]. Artificial intelligence is a rapidly developing technology that has applications in a wide variety of professions and areas of life. There have been recent breakthroughs in the pharmaceutical sector, employing this potent technology to address some of the most pressing issues confronting the sector. What we call “artificial intelligence” in the pharmaceutical industry is simply algorithms programmed to perform jobs that have always required human brainpower. The application of AI in the pharmaceutical and biotechnology industries in the past few years has revolutionized numerous aspects of drug discovery, disease treatment, and other related fields [3]. This conservative field has placed a premium on developing small size pharmaceuticals that are stable, effective in achieving therapeutic goals, and tolerable for a wide range of users [2,3]. Chemists use multimodal archives to systematically screen molecular variations for newly discovered molecules with promising properties that could be used in the field of healthcare. This is the most effective method in the research and development of medicines [4]. This method, however, is running out of steam as a means of meeting the rising demands of the healthcare business for innovative pharmaceuticals. This strategy has had little success in the research and development of new medications. To start, a great deal of research has already been conducted on potential synthetic replacements to small-size molecule therapies, for example, a beta-lactam ring containing antibiotics comes from penicillin, but there are only a handful of promising new possibilities for this development. In addition, exceptionally stable and powerful compounds are already available for a variety of therapeutic applications, setting a bar that is difficult for future molecules to meet [5]. The next issue is that, aside from a handful of specific uses or uncommon diseases, developing unique compounds is becoming less financially viable owing to an increasingly difficult route through clinical trials and intense rivalry from generic businesses [6].

**Figure 1:**
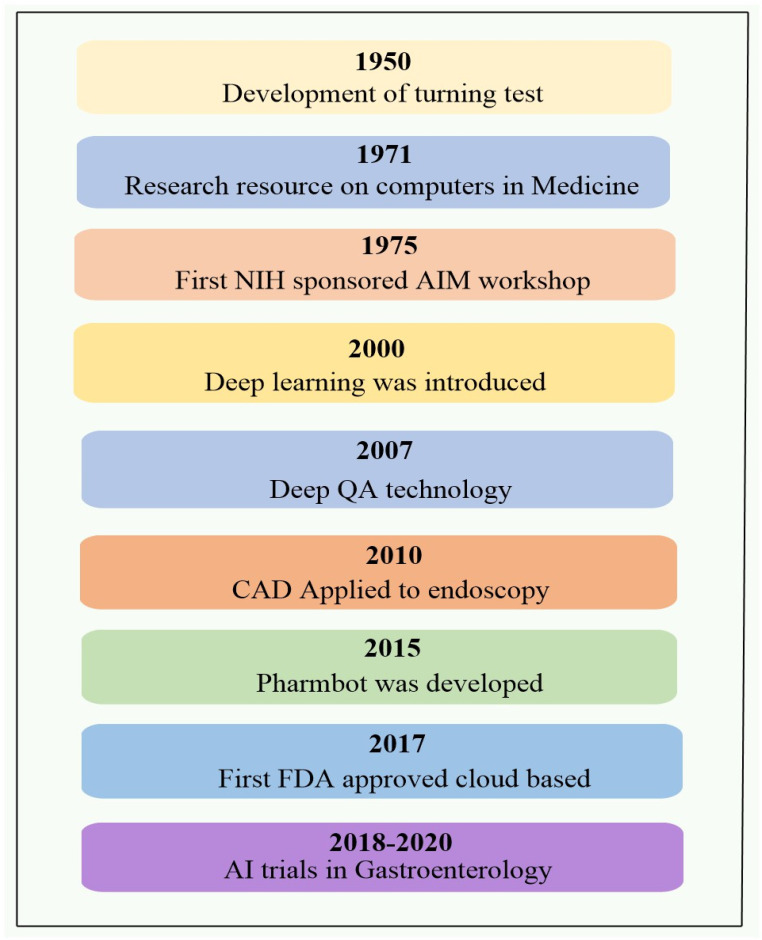
History of artificial intelligence in healthcare.

The following is an overview of AI’s medical application history: In 1950, AI made its first significant contribution to the medical field while working on shifting tests. The significance of computational intelligence in the medical field further came to light in 1975 with the development of a prototype study on computers in the field of medicine. Artificial intelligence’s reach in healthcare has grown since the advent of DeepQA software in 2007 and there have been profound learning advancements in the ensuing years. Additionally, endoscopy initially used CAD in 2010, and in 2015, the very new software Pharmbot was created. A watershed moment in the introduction of AI to healthcare occurred in 2017 with the launch of a new application named cloud-based DL, which received FDA approval. Multiple artificial intelligence trials in gastroenterology occurred between 2018 and 2020. A dramatic shift in how the pharmaceutical sector manages its supply chains has been made possible as a result of the use of AI (Figure 1).

**Figure 2:**
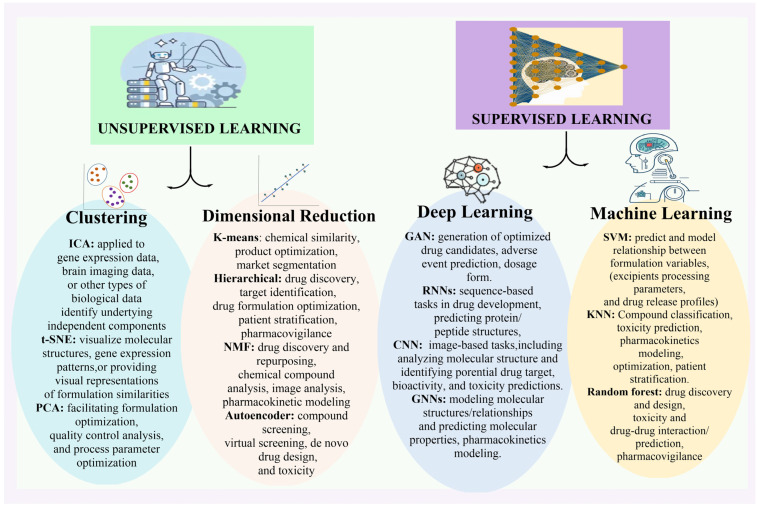
Varieties of AI machine learning algorithms, and both regular and free of control, with an emphasis on pharmaceuticals.

Furthermore, a great deal of artificial intelligence research that has been going on over a few decades has been synthesized with the objective of addressing a wide range of distribution network problems [7,8] (Figure 2). There has also been some investigation into the potential of multiple AI tools to improve various steps in the development of medicinal products. Table 1 provides a description of some of the most popular AI tools used in this field.

## 2. AI for Drug Discovery

**Figure 3:**
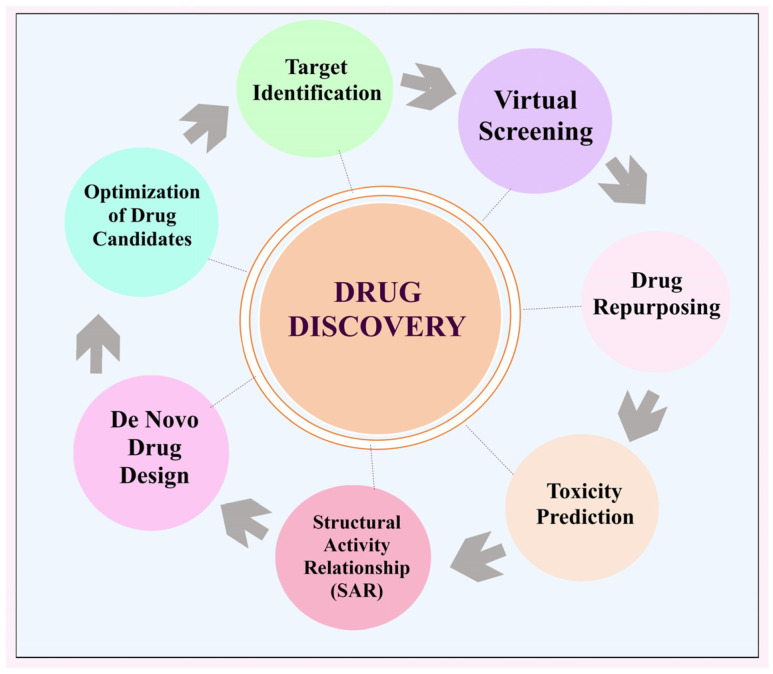
Process of Drug discovery with the help of AI.

AI has revolutionized drug research and discovery in numerous ways. Some of the key contributions of AI in this domains are illustrated in Figure 3.

### 2.1. Recognising Objectives

Promising biological targets can be identified by AI systems through the analysis of multiple data sources, including genomic, proteomic structural and function data. Artificial intelligence (AI) aids in the development of disease-modifying pharmaceuticals by revealing cellular processes and its domains linked with disease [23].

### 2.2. Screening via Simulation

Through the use of AI, massive chemical libraries may be efficiently screened to find potential medication candidates with a high probability of interacting with a particular receptor or domain. Artificial intelligence (AI) enables scientists to save energy as well as time by projecting binding sites and modelling interactions between chemicals, which helps them, focus as well as elect a suitable molecules for field trials [24].

### 2.3. Relationship Between Structure and Activity (SAR)

Artificial intelligence simulations can represent the relationship among a molecular structure and its biological action of compounds. Because of this, scientists are able to improve their therapeutic ideas by creating compounds with better pharmacokinetic characteristics, specificity, and efficacy [25].

### 2.4. Novel Drug Formulation

Machine learning together with generative models enable AI systems to suggest new chemical compounds that resemble drugs. Expanding the chemical universe and aiding in the discovery of novel medication, AI learns through chemical archives and data obtained from experiments [26].

### 2.5. Improvement of Potential Medicines

By considering pharmacokinetic factors, drug safety parameters, and its efficacy into account, AI systems can evaluate and improve drug optimisation. By doing so, scientists are able to improve the efficacy of therapeutic compounds while reducing the likelihood of adverse consequences.

### 2.6. Repurposing Pharmaceuticals

By analysing massive amounts of biomedical facts, AI may discover current medications that show promise as treatments for various illnesses. Artificial intelligence improves and speeds up the process of drug discovery by finding new uses for already-approved medications [27].

### 2.7. Predicting Toxic Effects

**Figure 4:**
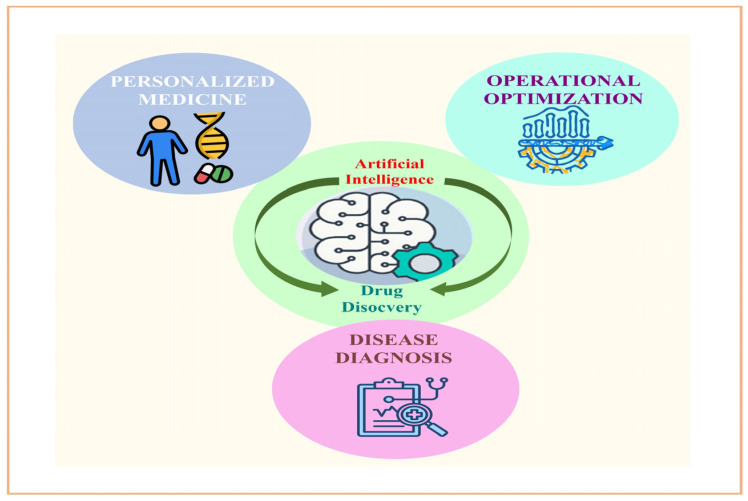
AI in drug discovery.

Through the analysis of molecules and properties, AI systems are able to forecast the toxic effects of drugs. By analysing toxicological datasets, artificial intelligence can detect potentially dangerous structural features or predict negative consequences. By doing so, they can reduce the likelihood of adverse reactions in clinical studies and prioritise the use of preferable drugs. The use of artificial intelligence (AI) in pharmaceutical R&D has the ability to improve the efficiency and effectiveness of medicine discovery, optimisation, including design processes [28]. The pharmaceutical industry, for instance, makes use of in-silico target fishing technology (TF) to anticipate biological targets from chemical structures. The biologically annotated data found in the library of chemical provides the basis for the provision of such data. In addition, by data extraction as well as docking of the chemical composition employed to investigate the mode of action [29]. Using artificial intelligence and cheminformatics, the target fishing technique had been applied to the medication development process. The correct analysis of complex frameworks along with the development of innovative medicinal components towards effective therapy of complicated diseases can both be aided by both of these. There are typical expansive drug discovery processes that are adopted by various companies, expensive because there is many complex steps like protein selection and its targeting and its mechanism of action. The use of TF expedited lowers the overall cost of experimental of medication development. The TF approach is often employed to investigate the phytopharmacology of the given sample and conduct similarity checks monthly. Sorting points from data according to the resemblance of data and pharmacological targets forms the basis of this analytical and proteomics database approach. The target based method of drug discovery also makes use of AI for the purpose of predicting possible risks and toxicities. The TF helps to discover and develop drugs by identifying and picking out new targets, predicting its phytopharmacological characteristics, and side effects in the management of new drug molecule. Methadone, loperamide, along with emetine are three drugs that were well profiled and by identifying their targets through this technique [30]. Evidently, many innovations that might ultimately be utilised in the production of pharmaceutical items have been developed as a result of recent advancements in artificial intelligence. During this time of intensifying competition, the actual winners will be those whoever can seize and use the advancement in technology strategically. Implementing potential is challenging since, in areas where implementations are currently successful, there is an opportunity to increase efficiency while simultaneously improving quality and uniformity [5] (Figure 4).

### 2.8. Artificial Intelligence Methods for Pharmaceutical Research

**Figure 5:**
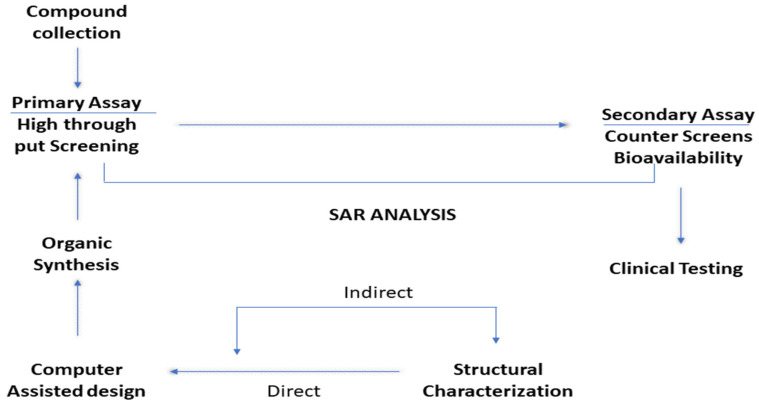
Approaches in drug discovery.

Using results from multiple sources is where the drug development process starts that involves models run on computers, data from earlier reports, fragment assessment, and the use of high-throughput screening [1,24]. Figure 5 shows the schematic depiction of drug discovery approach. Organic manufacturing of therapeutic compounds follows structural characterisation, which can be examined by using computer-assisted design either direct or indirect. The methods of induction and deduction are used interchangeably in the drug discovery processb which leads to optimised lead molecules [1]. Automation of certain inductive–deductive cycle steps reduces uncertainty as well as error, improving drug discovery. Researchers in the pharmaceutical and biotech sectors use artificial intelligence tools like “NVIDIA DGX-1” to sift through mountains of the patents and genetic data for any kind of useful findings. Humans can’t process all of the data that exists in order to further scientific knowledge. Supercomputers powered by artificial intelligence can aquire and process the data for identifying purposes [9].

Artificial Intelligence (AI) is transforming pharmaceutical research by enhancing drug discovery, design, and delivery. AI-driven models efficiently analyze vast biological and chemical datasets, accelerating drug development while reducing costs [31,32].

One of the earliest AI applications in pharmaceutical research involved chemical space exploration, which identified potential drug candidates from vast molecular libraries [33]. Computational techniques further improved drug discovery by aiding in target identification, where gene-disease association data helped predict novel therapeutic targets [34]. The integration of AI in computational drug discovery further streamlined the identification of promising molecules, enabling structure-based drug design [35].

AI-based de novo drug design emerged as a powerful approach, with tools like LigBuilder 2 generating novel drug-like molecules through rational design techniques [36]. Advancements in machine learning and deep learning have further refined AI applications, enabling predictive modeling for drug interactions and molecular properties [37]. Recent innovations in AI-driven drug delivery systems optimize pharmacokinetics, ensuring precise drug release and targeted therapy [31].

Further breakthroughs in AI-generated drug design introduced generative models, such as autoencoders, to create bioactive molecules with high specificity and efficacy [38,39]. Additionally, AI has played a crucial role in automated molecular design, where advanced deep learning techniques produce optimized small molecules for therapeutic use [40].

By integrating AI into pharmaceutical research, scientists can reduce drug development timelines while improving drug efficacy and safety. As AI technology continues to advance, its impact on pharmaceutical innovation will only expand [41].

Artificial intelligence’s potential uses in the pharmaceutical industry center on exploring the pharmaceutical space [1,42]. Since the target compounds may be computationally iterated, the realm of chemicals provides a platform for novel discovery [43]. Artificial learning associated with predictive techniques also aids in the discovery of useful compounds that are particular to domains [44]. The most challenging aspect of the overall procedure is choosing a novel medication molecule among a significant quantity of pharmacologically active components [45]. De novo design requires research in organic chemistry to synthesise in vitro compounds and simulated screening to substitute their biological as well as biochemistry testing to determine toxicity [46,47,48,49]. Drug discovery involves novel design to create new compounds that are active in the absence of standard compounds [47]. Molecular modelling is acquiring future artificial intelligence. For it, you can find a variety of computer programme. While this design isn’t practical for the discovery of drugs, it is related to creating components with challenging synthesis [50]. While iterative neural network designs have found use in entirely novel approach they were initially developed for use in NLP. As the molecular structures are encoded as a series of letters by SMILES strings, recursive neural networks are employed for generating the chemical structures of molecules. In order to train NLP with a larger number of molecules from existing pool, the syntax of SMILES sequences is conveyed to the networks (e.g., ChEMBL). For a large percentage of real SMILES strings, and NLP can process them [49]. Making more modern peptide sequences also makes use of this type of technique [51]. To help the produced molecule of a chemical exhibit the desired properties, reinforcement learning is also employed [52]. Learning through transfer is another helpful method to generate newer chemical compounds processing the correct biological property. There are two stages to this plan. First, we teach the network to understand SMILES grammar. The second stage is to keep practicing with combinations with the right properties. Even just a few more training phases can help create novel compounds by allowing them access to the same chemical domain that is present in standard compounds. In one study, these methods synthesized five compounds, with four chemicals exhibiting conformational action against a particular receptor/cells [47]. An intriguing technique in artificial intelligence called a “Variational Autoencoder” employs two pathways of neural networks first: the encoder pathway and the second is decoder pathway networks. The result is a continuous vector with real values extracted from the space of latent values. When it comes to chemical arrangements, encoder systems convert it into them; on the other hand, decoder systems perform the exact opposite [53]. After that, you can receive a molecular passage with improved target qualities.48 When compared to the ‘Variational Autoencoder’, the other software, ‘Adversarial Autoencoder’, is capable of generating much more realistic structures of molecules. One use of generative adversarial networks (GANs) is for developing medicinal compounds. The software can generate images that seem as good as the genuine thing from a written description. Using GAN, Kadurin et al. (2017) proposed chemicals with anticancer properties [54]. This technology may produce new data based on existing data or use imagination. Even the latest AI technologies can now solve even the most intractable problems [41]. These type of new tools helps scientists and other investigators find medicinal products that show promise and evaluate them for effectiveness and safety, followed by choosing which people to enroll in research studies [1,24]. Drug delivery benefits from AI’s capacity to rank compounds according to their synthesis and the establishment of practical tools that have proven effective for determining the most suitable procedures for synthesis [42]. we can see a compilation of some of the most useful AI-powered computational resources for the pharmaceutical industry from Table 2.

## 3. Artificial Intelligence in Individualised Computerised Therapy

**Figure 6:**
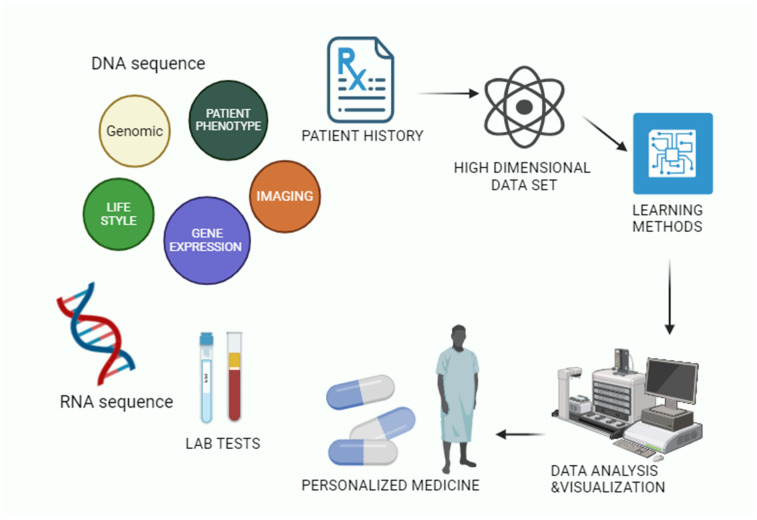
AI in acquiring and analyzing data of a patient in personalizing the treatment.

In order to aid in illness evaluation, therapy, and prevention, AI may be able to extract useful relationships from raw information sheets. The recently established field of algorithmic knowledge employs a number of cutting-edge methods that might find utility in virtually every area of medicine. The complexity of solving the complicated healthcare issues lies in gathering, analysing, and using huge amounts of data (Figure 6). With the advancement of technology such as AI, doctors have been able to tackle previously intractable clinical issues. Helping medical professionals manage data are technologies like artificial neural networks (ANNs), hybrid intelligence systems (HIS), evolutionary computation systems (ECS), and fuzzy expert systems (FES) [46]. The artificial neural network, or ANN, is an architecture that mimics the way the brain and spinal cord works [47]. In order to manage information in parallel, a chain of linked processors on computers known as neurons can be utilised. In order to create the initial fabricated neuron, a type of binary sensitivity function was utilised. There are numerically weighted connections between every single neuron [48].

Identifying a contagious disease is usually an afterthought, and stopping its dissemination calls for constant monitoring and analysis. Therefore, taking swift action based on reliable information has a substantial social and economic impact on people’s lives all over the world [49]. One major advantage of using AI for medical purposes is the potential for advancements in areas such as collecting and analysing information, as well as the development of surgical robots. In this article, we will go over the several ways AI may be used in medical treatment, the signs and symptoms of diseases, the challenges with diagnosis, and a framework for recognising diseases that uses AI [50].

### 3.1. AI-Based Illness Prediction Modelling Framework

**Figure 7:**
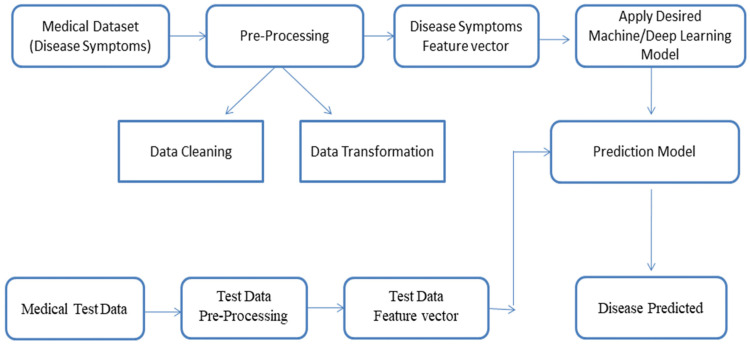
System architecture for illness detection.

The capacity of a computer to mimic human learning processes, such as pattern recognition in challenging situations and picture identification, is known as artificial intelligence (AI). Using AI in medical settings alters the way data is created, evaluated, and used to improve care for patients [51]. The first and most basic step in designing an arrangement is called system planning. It encompasses the framework’s perspectives, and trajectory, and the way the framework operates under explicit constraints. When the consumer has an established understanding of a framework, they’ll be better able to understand its constraints. The illness assessment model, which makes use of practical machines and deep neural classification techniques, is illustrated graphically in Figure 7. Prior to being handled by the computation, actual-life information necessitates maintenance along with pre-preparation [52]. Reasonable explanations aside, actual information frequently includes errors related to the used metrics, but these errors are unable to be practiced. Data preparation, then, is taking this raw data, cycling it to remove mistakes and unnecessary examinations. There is a series of phases that information goes through before it is handled [53,54]. There are a variety of approaches to data cleansing. Information acquisition, like filling in blanks, and information reduction, like removing commas and other cryptic characters, constitute a single part of these tactics. Data is combined from multiple sources in an information osmosis process. After then, any combination of errors is corrected, and the issues are promptly addressed. Changes to Data: This step involves standardising data based around the provided calculation. There are multiple approaches to implementing information standardisation [55]. Since the goal of the majority of mining algorithms is to obtain ideal data, this step is required. Subsequently, information is shared and refined. Data Reduction: This step in the process focuses on reducing the data to a more useful level. Collecting information and assessment: Data collecting is divided into two sections: data preparation and data testing. In order to evaluate the real data samples, preliminary information is used [56]. It is common practice to copy data collected during experiments from a comparable informative index, which is comparable to the data essential to preparation and assessment. Quickly testing the framework’s accuracy follows model pre-handling. The organised model is highly effective in disease prediction because it employs analytical showing methodologies to determine the likelihood of an event by given parameters. Based on the user’s data indicators and past decisions, it attempts to put itself in their shoes [57,58].

### 3.2. Diagnostic Imaging in Medicine

Assigning techniques that generate images of the interior of an individual’s body is what diagnostic imaging is all about. Clinical applications of the technique include taking images of the anatomy for the goal of diagnosing, studying, or revealing injuries, fractures as well as and abnormalities [59]. The results of computed tomography (CT) are excellent illustrations of useful confirmatory imaging which facilitates precise diagnosis, mediation, and assessment of injuries and problems that professionals in the field routinely address [60]. Table 3 shows that there are other considerations that illustrates the excessive application of imaging, such as X-rays as well as magnetic resonance imaging (MRI), for complex and demanding tasks.

### 3.3. Optimization of Pharmaceutical Products

A costly path for commercialization, poor success in clinical trials, and lengthy processes are some of the challenges that small-molecule medicines face throughout their research and development cycles. Several reasons are at play here, such as the market’s saturation, challenge of getting regulatory approval for novel compounds, and even the willingness to spend in advanced countries as well as developing one’s [61,62]. The transition from basic research into clinical investigations is notoriously challenging in the process of drug discovery. Despite an explosion in the amount of high-quality research materials, a lot of investigators still lack the skills necessary to properly evaluate and use these resources through their particular approaches and projects. Machine learning along with domain-driven “weak” AI offers new possibilities for the creation of small size novel drug. Machine learning innovations, which can be seen as instances of weak AI, have made tremendous stride when it comes to their fundamental algorithms and their practical applications. Therefore, our research will center on the parts of such exciting area that are currently showing their worth, as well as the advancements that hold the greatest prospect for the forthcoming phase of artificial intelligence in medication creation [63].

## 4. AI-Based Drug Delivery System

The arrangement of systems for drug delivery is often associated with some drawbacks, such as the need to anticipate the interaction between formulation parameters and reactions [64]. The treatment effects and unforeseen events are also connected to this. Important considerations for the development of various smart pharmaceutical release systems include drug stability, selective release, dosage modification on the demand, and even the rate of release of drug [65]. The appropriate algorithms are helpful for managing the amount and duration of drug release by monitoring their own with the help of such devices. Hence, AI methods can be helpful in predicting how well drug delivery dosage forms will work and what drugs they will contain. Solid form of dispersion of Carbamazepine utilizing poloxamer 188 as well as Soluplus^®^ developed by combining ANN modeling [66]. The goal of making carbamazepine in solid form intended to make it more soluble and to speed up the rate at which it dissolved. To optimise medication dissolution, an ANN approach known as feed-forward back propagation, with the multivariate sigmoid function that analysed the factors associated with dissolution [67]. For medication solid form dispersions, the use of ANNs-deriven approaches led to the accurate prediction of dissolving characteristics and physical stability over the long term [68].

### 4.1. Microemulsions and Emulsions

The production of stable oil in water emulsions has also made use of ANNs in the composition process. In this part, we looked at ways to optimize the proportion of lipid alcohols in oil/water emulsions. Time as well as the proportion of lauryl alcohol is the two unreliable parameters considered in this study. Size of the droplets, thickness, conductance, and zeta potential are the reliable variables. The validation procedure revealed a high degree of agreement between the experimental data and the values predicted by ANNs [69]. Microemulsion has designed by ANNs, allowing for easy analysis of accuracy predictions that based on the formulation [70]. The combination of genetic programming and adaptive artificial neural networks has also allowed for the highly accurate prediction of microemulsion type and internal structural aspects. A further research investigation used ANN simulations to predict how to manufacture microemulsions that stable orally containing antitubercular medications such as isoniazid and rifampicin [71]. Evaluation and assessment of the models of ANN are conducted using data derived from the pseudo-ternary phase triangle-diagrams that showed the combination of surfactant with oily components of emulsion.

### 4.2. Tablets

The designers have used dynamic along with static ANNs to model the disintegration characteristics of various matrix-type tablets [72]. These ANN-based models were optimized by employing the genetic algorithms optimizer tool and Monte Carlo simulations. Elman dynamic neural networks as well as decision trees are employed by the researcher to accurately forecast the solubility characteristics of lipophillic and hydrophilic matrix tablets with a controlled release of therapeutics. Researchers used a perceptron with multiple layers by feedforward back propagation technology, to create matrix pills that release drug like metformin HCl, a hypoglycemic drug, over a prolonged period of time [73]. Matrix tablets of drug Nimodipine have been modified for controlled-release administration in a different research that included ANNs in the formulation process [74]. Glipizide osmotic pump pills were developed using a methodology that combines an artificial neural network (ANN)-based concept with a statistical refinement procedure [75]. An analysis was conducted using artificial neural networks (ANNs) in order to optimize and confirm the various process as well as formulation aspects. To optimize the dosage form of drug, isradipine osmotic pills, a combination of response surface methodology (RSM) with artificial neural network (ANN) analysis was used [76].

### 4.3. Beads, Microparticles, and Nanoparticles (Multiparticulates)

Multiparticulate, verapamil beads were created with the use of computer-aided design and chemical software [77]. The experimental study of verapamil release from the beads was examined, along with the effects of process and formulating parameters. When compared to the anticipated outcomes produced through ANN approach, in-vitro releasing pattern of verapamil beads were turned out to have a satisfactory concordance. ANN modeling has been employed to evaluate process factors influencing enzyme papain entrapment in the beads of alginate for stability improvement and release on a particular site [78]. Floating microspheres of aspirin in alginate were optimized using both RSM and ANN, but ANN predicted a better pattern of aspirin release than RSM [79]. Microspheres in polymeric form were developed using the ANN technique and factorial design for multivariate approaches. The study indicated that ANN performed better, was unbiased, and predicted more accurately than the factorial method. The study’s results demonstrated that the ANN model showed better fitting abilities with comparatively less biased and more accurate predictability than the factorial model.

### 4.4. Application of AI Tools in the Design of Dosage Forms

**Figure 8:**
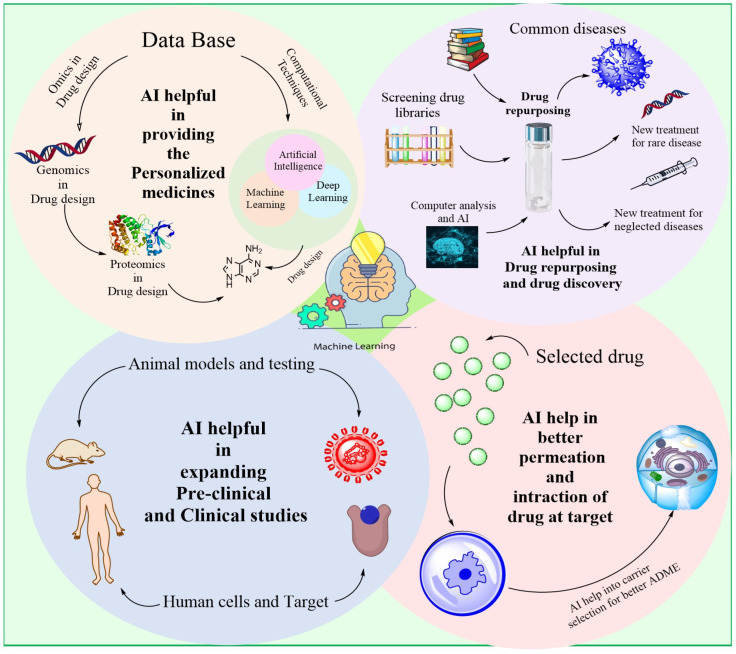
AI can improve AI can improve the design of nanosystem, broaden the drug assessment modelling, improve features and aspects for choosing a particular design, exploration, and recycling. Drug penetration, modelling, cell targets, etc. assist explain transmembrane connection with the modelled human milieu.

To comprehend the effects of medication administration, the machinery of the human body is segmented. Organized according to cellular membranes, the sections are further reduced in complexity. Physiological compartments rely on physicochemical obstacles which can be applied according to the route of medication administration within the organism. The rate of penetration depending on the way of administration is an essential requirement for successful surveillance of drug distribution systems. The gastrointestinal epithelial cells should be permeable for orally supplied medicine to reach the stomach milieu [80]. The drug’s subsequent diffusion into circulation depends on that stage. The medicine is transported towards its target region, during the procedure of distribution [81]. Biochemical barriers, whether they are passive or active present, allow the majority of medication absorption. In diffusion that is passive, the chemical properties of the medication are the basis. There is some discrepancy between the computational model predictions and the real findings when it comes to computational examination of distribution of drug [82]. The drug’s bioavailability and how it interacts with its biological constituents greatly influence the drug’s trajectory through the body. The biological and chemical properties of the medicine regulate this procedure. Passive permeation seems ineffective with numerous tiny molecules and physiologically active substances, necessitating a specialized medication delivery mechanism. Transport through membranes is the driving force of active permeation, which is dependent on intricate interactions between organisms. Computational and methodical modeling methodologies involving several particular variables are necessary for investigating this complicated process. A new computer model is employed to evaluate medication delivery system pharmacokinetic attributes [83]. Predictability of preclinical models is a critical gap in pharmacy research and development. Even for complicated in silico designs, the chosen parameters form the basis of the forecasting assumption. Figure 8 shows that the simulated environment is able to better assess all of these cases, which are associated with interactions of medicines with membranes. With the help of AI, we can study and evaluate this simulated world better [84]. Artificial intelligence (AI) offers high-tech tools for analyzing data with multiple layers. A deeper comprehension of the study units will result from the comprehensive analysis. At each stage of the research process, numerous aspects, including goals coming, simulation, and modification, are used to establish the optimal results through the methodical application of the model and factor analysis. AI has the potential to automate every one of these duties, allowing for more accurate predictions and more frequent data refining. Based on the database type (system biology), it is clear that a thorough comprehension of the interaction between drug and biological processes is crucial for improved artificial intelligence instruction within a biological setting. Neural networks based on artificial intelligence are just one of many cutting-edge AI tools that can be used to conduct pharmacokinetic investigations. Additionally, AI provides a plethora of datasets, including chemical, physical as well as genomic information, which aid in comprehending medication interactions and studying the intricate fundamental activities of compounds within systems. To improve our knowledge of the accumulation and toxicity of drugs, we also use several of these approaches to examine how the drug distribution affects its absorption and metabolism [85]. The development of quality and important features, as well as the assessment of their effects on clinical research prior to actual attempts, is a common aspect of many innovative drug distribution approaches [86,87].

## 5. Ai-Powered Resources for the Creation of Biologics

Modern drugs, immune-modulating agents, nucleic acid products, and peptides can all be aided by AI [88]. Protein molecules with certain properties could be more easily created with the use of artificial intelligence [89]. Through the analysis of extensive amounts of data pertaining to the nature and activity of proteins, artificial intelligence (AI) models have the potential to generate therapeutic sequences that exhibit affinity for attachment, enhanced stability and immunogenicity. Because of this, personalised biologics can be developed, which greatly enhances their efficacy and safety [90]. Using clinical, information of protein, and genomic data, AI algorithms can identify potential treatment targets. By identifying disease objectives, AI aids in the development of biological products of protein that modify pathways in the body or specific proteins which trigger illness [91]. Folding of proteins can be predicted by AI models using patterns of amino acids. Optimal biologics as well as an improved comprehension of protein structure and function are both made possible by folding proteins. To aid in the design of stable and functional biologics, deep learning and molecular dynamics simulations can predict folding protein sequences (Figure 6) [92]. AI systems can foretell the degree of binding propensity between proteins and their target molecules. By training on massive protein-peptide or protein-protein datasets, AI models may relyably forecast binding strength. This enhances the efficacy of treatments by selecting or developing biologics that bind to specific targets with great selectivity and adherence [93]. AI has the potential to enhance the formulation of biologics involving proteins and peptides. The efficacy and overall quality of biologics are impacted by formulation variables, resilience and aggregation tendency. By studying physical and chemical features of proteins, manufacturing processes, and composition, AI algorithms optimise physiologic stability, duration of storage, and composition [94]. AI systems can foretell the potential biological toxicity of proteins and peptides. Using toxicological information for training, artificial intelligence (AI) programmes may analyse SAR in order to anticipate adverse effects that affect immunity. By doing so, they can identify potentially dangerous segments or patterns and modify them [95,96,97,98,99]. C clinical trials on biopharmaceutical based on protein are making use of AI to optimise their execution. Clinical trials on biopharmaceutical based on protein are making use of AI to optimise their execution [100]. Machine learning systems can analyse patient records, disease characteristics, and therapeutic outcomes to refine trial protocols and make accurate predictions about patient reactions [101,102]. Exosomes, CRISPR/Cas9 and CAR T-cell therapy are three areas where artificial intelligence (AI) can greatly improve research, evaluations, and treatments [103].

## 6. AI for Epidemic and Pandemic Forecasting

The potential for pandemics to cause illness and death is limitless. A number of pandemics have broken out over the world, including the cholera, Black Death, influenza, the Spanish flu, AIDS, and even COVID-19, each of one have the potential to disrupt the economy and society [104]. Early diagnosis and effective control of illnesses alleviate strain on people’s health as well as on monetary; these spheres are highly interdependent. Surveillance is crucial for detection in advance [105]. Massive amounts of time, energy, and money are required for ongoing monitoring. Epidemic as well as pandemic prediction is difficult in practice. But now we can study the spread of terrible diseases thanks to scientific breakthroughs. To do monitoring while making the most of available resources, AI becomes the way to go. Many areas associated with healthcare are starting to use ML as well as extensive learning because it’s more efficient than recruiting and hiring [106]. The intricacy of epidemiological simulations makes their development a continuing challenge. Upcoming models for epidemic prediction have begun integrating ML [107]. During pandemics or epidemics, AI is utilised for the following tasks: identification, prevention, action, and rehabilitation. Particularly in light of the current COVID-19 pandemic, its usage in estimation, monitoring, and data collection is expanding rapidly within the realm of preventive [108]. Epidemics of Influenza are notoriously difficult to forecast because of factors such as the fact that they can change their peak at any time and have recurrent peaks. Even in regions with unpredictable seasonal cold, a precise prediction is within reach through the help of SAAIM (self-adaptive AI model) [109]. As an example, a recent study in Taiwan found that flu may be accurately predicted using machine learning along with ensemble methodologies [110]. A 90% accuracy rate in influenza forecasts was achieved employing the MSDII-FFNN model, which is a machine learning feed-forward propagation neural network [111]. Both the United States and Australia have begun using artificial intelligence anonymized mobility maps (AMM) to forecast the spread of influenza. Even when people travel across state lines, AMM may still use this information to predict when epidemics would strike by grouping information collected from smartphones together [112]. Ebola continues to be a problem throughout Africa. One of the many methods used to forecast the spread of Ebola has been a sort of hybrid neural network created by Umang Soni et al., that, when trained with arbitrary forest as its classification algorithm, achieves a perfect accuracy rate of 100% [113]. Reliable predictions for the dissemination have been made using simulations that incorporate computer learning and virtual societies. In a case study, researchers have investigated and forecasted the eventual result of the outbreak of Ebola using a virtual model in Beijing [114]. During the year of 2015, Zika pandemic, it was extremely difficult to distribute surveillance resources because accurate predictions were not available. The subsequent spread prediction was based on an algorithm of a dynamic neural network system. During the early stages of the outbreak, this adaptable framework for predictive models was useful and dependable [115]. In order to keep tabs on the number of mosquitoes in the region, the Zika deployed a mobile app that used artificial intelligence neural networks for preliminary identification [116]. Attention has been drawn to vaccine-derived poliovirus (VDPV) monitoring because to its results. A VDPV epidemic can be predicted using hybrid form of machine learning that combines the whale optimisation algorithm (WOA) using random vector functional link (RVFL) networks [117]. The use of ML in AIDS management strategies could help identify those who could benefit from prevention prior to exposure [118]. Dengue fever is common in regions with temprate climates. When it comes to monitoring outbreaks of dengue in China, the ML method supported vector regression, or SVR, can do so with almost no mistake at all [119]. The best dengue predictor in Malaysia was a linear kernel ML support vector model (SVM) [110], and to forecast dengue outbreaks, researchers used Bayesian network ML approaches [111]. The total efficacy is near to 100%, when the artificial neural network (ANN) is used for quick identification from given data of TB wary. In doing so, we may more easily gauge the disease’s general spread and put a stop to it quickly [112]. One convolutional neural network (CNN) model, tuberculosis AI (TB-AI), demonstrated sensitivity of 97.94% when it came to identifying tuberculosis bacteria [113]. Yellow fever was diagnosed with an 88% prediction accuracy using a Multilayer Perceptron Neural Network Classifier (MPNN) that took seven behavioural signs into account [114]. Global panic ensued as the COVID-19 pandemic broke out. To predict the COVID-19, we used a stacked auto-encoder model, which was prompted by artificial intelligence. Fuzzy rules and deep learning’s Composite Monte Carlo (CMC) were useful for predictions of the COVID-19 epidemic and making choices [115]. With a polynomial neural network (PNN) and corrective feedback (CF), forecasts can be made with almost no error at all [116]. Among China’s deep neural networks, CNN stands out for its pinpoint accuracy in making predictions [117]. Predictions of COVID-19 in Switzerland are based on ’Enerpol’, an AI model with combination of Big Data [118]. Statistical as well as deep learning methods including ARIMA, MLP, FNN, and LSTM were utilised to study the COVID-19 changing pattern. It is possible that the generated data can serve as a valuable resource towards the COVID-19 estimate [119,120].

## 7. Intelligent Systems for Pharmacokinetics and Pharmacodynamics

**Figure 9:**
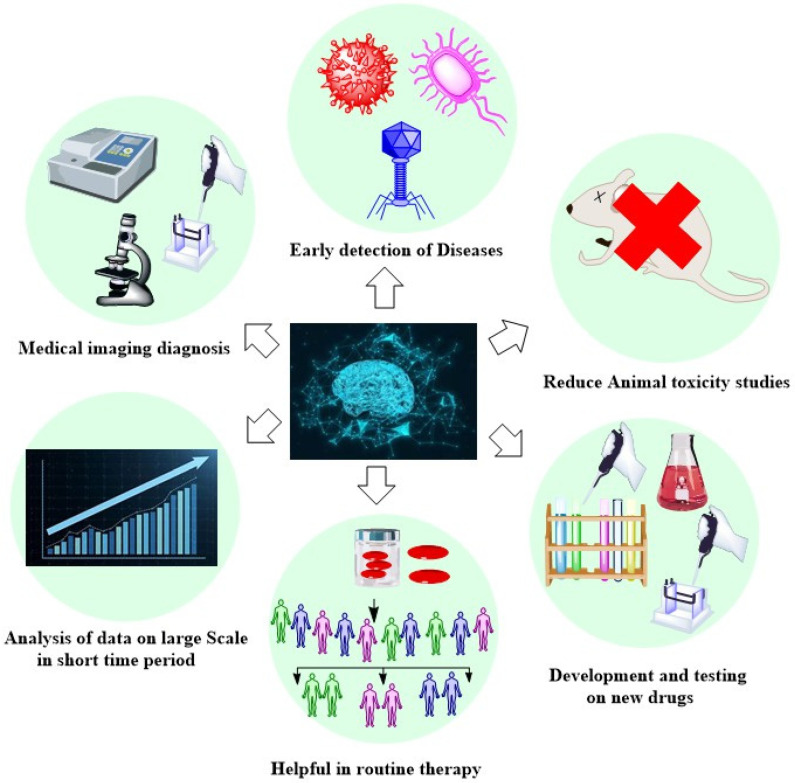
Role of AI in PKPD studies. Pharmacokinetic studies include absorption (A), distribution (D), metabolism (M), and excretion (E) studies.

Pharmaceutical discovering things, preclinical research, experimental studies, along with regulatory clearance are all parts of the lengthy and intricate process of developing drugs. The ideal dose, route of administration, and the effectiveness of a medicine in the human body are determined by pharmacokinetics and pharmacodynamics, which are essential parts of the development of drugs [121]. When studying the pharmacokinetics as well as the pharmacodynamics of a medicine, the conventional experimental approaches can be costly and lengthy, and they don’t guarantee that the drugs will be effective and safe [122,123]. Research on the relationship between pharmacokinetics and pharmacodynamics, relied on experimental approaches like animal research and clinical studies. Ethical considerations, sample size, and variability between individuals are some of the major obstacles to these methodologies. In addition, the pharmacokinetic along with pharmacodynamic outcomes of drugs for human beings may not necessarily be predictable from these trials. To circumvent these restrictions, computer models and AI-based approaches have been created to forecast the pharmacokinetics and also pharmacodynamics of drugs in a more efficient, economical, and precise way [124,125]. Drug discovery, drug kinetics, and pharmacodynamics are three areas where AI has demonstrated great promise (Figure 9) [126]. Predicting and optimising medication pharmacokinetics with pharmacodynamics has never been easier than with AI, thanks to the rise of sophisticated computing and artificial intelligence techniques. Artificial intelligence (AI) has the potential to revolutionise PKPD research and its therapeutic implications, despite the insurmountable obstacles posed by big data and trustworthy datasets [127,128,129].

The effect of the medicine on the target is a part of a pharmacodynamic investigation. It takes a lot of computations to fully comprehend the distribution and action of pharmacological compounds. A major mistake could result from a minor error, such as an overlooked dataset or incorrect calculation. The use of AI allows for the acceleration of complex computations even in the presence of incomplete datasets, leading to more accurate, quicker, and more affordable outcomes. It simplifies complex data into info graphics that anybody can comprehend and visualise, which could lead to the discovery of the problem’s origin. Reducing the number of mice needed for clinical trials is another benefit of this method, along with the ability to estimate the effect of many factors (enzymes, sick states, dosage variations, patient data, etc.) in various animals.

### 7.1. Parameters for Drug Release and Absorption Prediction

It is possible to anticipate the release of drugs and absorption characteristics with the use of models based on artificial intelligence. By analysing information from different medication delivery systems, AI algorithms can forecast how pharmaceuticals will be released into the body. AI models can predict drug release over time by considering the medication’s physicochemical features, composition, and delivery system release mechanism. Models powered by artificial intelligence may also foretell how medications will be released from various drug delivery devices, including inhalers, patches for the skin, and oral pills [130]. By taking into account elements like the solubility, accessibility, and composition properties of the drug, AI-based models can forecast the rate of absorption and effectiveness. By taking into account elements like the solubility, accessibility, and composition properties of the drug, models based on AI can forecast the rate of absorption and effectiveness. In conclusion, models powered by AI offer a potent resource for forecasting parameters related to drug dissolution and absorption. The models can improve the way drugs are delivered, aid in drug design decision-making, and optimise formulations for drugs by assessing different parameters and using artificial intelligence [131].

### 7.2. Estimation of Metabolism and Excretion Indices

By anticipating drug breakdown and elimination parameters, artificial intelligence (AI) models have demonstrated their utility in elucidating drug dynamics. By analysing the molecular makeup and physical and chemical characteristics of pharmaceuticals, AI algorithms are able to forecast their pathways of metabolism. Structure-associated features of particular metabolic transformations can be identified by AI models through training on extensive datasets containing known metabolism of drugs details. The aforementioned models facilitate the forecasting of prospective metabolites and offer valuable insights into the principal enzymes implicated in the chemical breakdown of drugs [132]. Estimating the metabolic fate of pharmaceuticals is possible through the calculation of the kinetics of enzymes including reaction rates alongside protein–substrate conversations, by models powered by AI. Machine learning algorithms are capable of evaluating the potential influence of metabolism on drug elimination and effectiveness through the consideration of drug–drug conversations, enzyme expression levels, and variation in genes. The provided data holds significant value in the optimisation of drug dosage regimens and the prediction of possible interactions between drugs [133]. In order to forecast the drug’s elimination rates, algorithms that use AI may examine physical and chemical features of the drug, including lipophilicity, ionisation, and molecular weight. This models can approximate the rate that drugs get eliminated via the body by undergoing training using databases that contain data regarding drug clearance routes. The data is of the utmost importance in establishing suitable dosage plans and guaranteeing the effectiveness and safety of the medication [134]. The interactions of drugs with transporters implicated in the processes of digestion, elimination, absorption, and distribution can be predicted by AI models. Through the examination of transporter traits and drug physicochemical properties, AI models are capable of evaluating the possibility of interactions among drugs or modifications in pharmacokinetics resulting from carrier-mediated effects. This information facilitates the comprehension of drug disposition and the enhancement of drug formulations [135,136,137,138,139]. Through the application of AI algorithms along with the examination of extensive datasets pertaining to drug metabolism as well as excretion, such models make a valuable contribution to the prediction of drug trajectory within the organism. They contribute to the optimisation of drug dosing, the identification of potential drug interactions, and the development of more effective and safer drugs. Table 4 shows the computer programs used for patient data collection for dosing and pharmacokinetic medeling. Moreover, artificial intelligence (AI) models empower pharmaceutical enterprises as well as scientists to rank drug candidates according to their anticipated metabolic processes and elimination profiles, thereby streamlining every aspect of developing new drugs.

## 8. Hospital Pharmacy AI

AI is utilised in hospital pharmacy-associated medical facilities for various purposes, including organizing medication dosages for individualised patients, choosing readily accessible or appropriate administration pathways, and formulating treatment policies [152].

(i) Health record upkeep: Preserving patients’ health records is an intricate responsibility. Data acquisition, storage, normalization, and tracing are simplified by implementing an AI system. Google’s Deep Mind health initiative, which Google created, aids in the rapid retrieval of medical records. Therefore, this endeavour contributes to the advancement and expediency of healthcare. This initiative provides support to the Moorfields Eye Hospital NHS in its endeavour to enhance eye treatment. (ii) Treatment strategy development: AI technology enables the creation of efficacious treatment plans [153]. In situations involving critical patient conditions that complicate the decision-making process of an appropriate treatment plan, the implementation of an AI system becomes imperative for situation management. The therapeutic plan recommended by this technology takes into account all prior information and reports, medical expertise, and other relevant factors. IBM Watson introduces a programme designed to assist oncologists. (iii) Supporting monotonous tasks: artificial intelligence also aids in identifying and detecting of diseases and disorders through studying repetitive duties like X-ray imaging, imaging, ECHO, ECG, Medical Sieve and radiology, an IBM-introduced algorithm, functions as a “cognitive assistant” with strong reasoning along with analytic capabilities. By integrating medical data via deep learning, a healthcare startup is essential for the enhancement of patient conditions. For each body portion, a specialized computer program is accessible and employed to diagnose particular disease conditions. Deep learning applies to the vast majority of imaging analysis categories, including X-ray CT scanning, ECHO, and ECG, among others. (iv) Healthcare support along with medication assistance: In recent times, the efficacy of machine learning in providing medication and medical assistance has been acknowledged. Molly, a simulated nurse developed by a startup, has an agreeable facial expression and voice. Its purpose is to assist patients in directing their own treatment and provide support for those with chronic conditions between doctor visits. An application that resides on the webcam of a smartphone, Ai Cure, observes patients and helps them manage their health issues. (v) Artificial intelligence contributes to improving the medical care system by accumulating and comparing data generated by societal consciousness algorithms. The extensive database maintained in healthcare systems comprises people’s medical records, treatment histories, routines, and lifestyles from birth to the present.

## 9. Applications of AI to Polypharmacology

As a result of the molecular underpinnings of numerous pathological processes, the ‘single-disease-many-targets’ concept has supplanted the ‘one-disease-one-targets’ concept in contemporary times. “A single disease, numerous targets” refers to the polypharmacology phenomenon [154]. A wide array of valuable databases are at one’s disposal, including but not limited to PubChem, ChEMBL, KEGG, ZINC, STITCH, PDB, Ligand Expo, Drug bank, Binding DB and Supertarget. These databases provide access to a multitude of significant and practical information about molecular processes, crystal structures, biological features, chemical characteristics, disease considerations, binding affinities, along with drug targets. AI also facilitates the identification of databases containing sketches of polypharmacological substances and molecules [155].

## 10. Benefits of Artificial Intelligence

The following are prospective benefits of AI technology: [152,153]

(i) Error minimization: Artificial intelligence aids in reducing errors and enhancing precision and dependability. Robots with intelligence are deployed for space exploration due to their durable metal construction and ability to withstand the hostile atmosphere of space. (ii) Difficult exploration: The mining sector demonstrates the utility of AI. In addition, the petroleum exploration industry employs it. Artificial intelligence systems can explore the ocean by eliminating human error. (iii) Practical Implementation: Artificial intelligence is highly advantageous in our routine activities and undertakings. Instances where GPS systems are widely utilised include lengthy drives. The installation of artificial intelligence on Android devices enables the prediction of user input. Additionally, it aids in the rectification of misspellings. (iv) Digitally assistants: In contemporary times, sophisticated organisations are implementing AI systems such as “avatars” to alleviate manual labour. The ’avatar’ is capable of making rational decisions devoid of any emotion. The impact of human feelings and thoughts on the efficacy of decision-making can be mitigated through the execution of machine intelligence. (v) Repetitive tasks: Generally, the human lifespan is limited to a single task at a time. Machines can execute tasks requiring multiple tasks at once and exhibit greater analytical speed than human beings. Different machine parameters, such as time and pace, are modifiable by the user’s specifications. (vi) Medical applications: With the assistance of AI programs, physicians can generally evaluate the health status of individuals and assess the potential adverse effects and other health hazards that may be linked to the medication. By utilizing AI programs such as a variety of artificial operation simulators (e.g., gastrointestinal experiments, heart models, brain simulations, and so forth), surgical trainees can acquire knowledge. (vii) No pauses: In contrast to individuals who can work eight hours a day with breaks, the programming of the machines enables them to operate continuously for extended periods without experiencing any form of distraction or monotony. (viii) Accelerate technological development: Artificial intelligence (AI) is extensively integrated into most cutting-edge technological advancements globally. The system can generate various computational modeling programs and strive to develop novel molecules. Additionally, AI is being implemented in the development of systems for delivering drugs. (ix) Risk-free: Working in hazardous environments, such as fire stations, entails significant potential for damage to personnel involved. The compromised components may be reparable if an error occurs with machine learning programs. (x) Assists: AI technology has assumed an alternative role by providing round-the-clock assistance to infants and elderly individuals. It can serve as an educational resource for all individuals. (xi) Functions without limitations: Machinery is not constrained by any restrictions. Dispassionate machines can produce with greater precision and efficiency than humans.

## 11. Drawbacks Associated with AI Technology

The significant drawbacks associated with AI technology tend to be as follows: [152,153].

Expensive: The introduction of AI results in enormous financial outlays. Sophisticated machine development, upkeep, and repair are extraordinarily economical. Significant time is invested by the Research and Development (R&D) section in the development of a single AI machine. Software applications for AI machines must be routinely updated. The a reinstallation and recuperation of the machine require significant financial investment and additional time.

No human duplication: Robots equipped with AI technology are renowned for their ability to think like humans and lack of sentiment, both of which contribute to the enhanced precision and impartiality with which they execute assigned tasks. When unanticipated issues arise, robots are incapable of rendering erroneous reports or making decisions.

Absence of improvement with training: Experience can enhance human resources. On the contrary, AI-enabled machines are incapable of gaining experience. They are incapable of distinguishing between individuals who are diligent and those who are unreliable.

Absence of originality: AI-powered machines fall short in both sensitivity and emotional intelligence. Individuals possess the faculties of vision, hearing, touch, and thought. They are capable of employing both their rationality and imaginative faculties. These characteristics are unattainable through the use of machinery.

Unemployment: Proliferating implementations of AI technology across industries could result in significant levels of joblessness. As a result of undesirable unemployment, employees may experience a decline in productivity and innovation.

## 12. The Constraints of AI Tools

Recent advances in artificial intelligence fascinate scientists, particularly when it comes to its implementation in pharmaceutical along with healthcare studies and service provision. Intelligent healthcare facilities along with healthcare facilities powered by AI, ML, when Big Data will shape the future of the healthcare industry. The pharmaceutical and biotechnology sectors are constantly advancing their technologies, and artificial intelligence will present a chance to reduce the time and expense of drug development.

## 13. The Significance of Explainability in Deep Learning Models, Particularly in the Medical Field

A wide variety of fields have witnessed rapid advancements in methodology based on Artificial Intelligence (AI) in recent decades. There have been numerous reports of strategies utilising ML and DL models in this quickly developing sector. The majority of these models are ‘Black-Box’ since they are sophisticated by design and do not provide any explanations for how they make decisions. Financial services, online shopping, healthcare, public safety, and other mission-critical application areas have a hard time implementing such models due to the difficulties in understanding them. As these AI models evolve at such a rapid pace, it becomes more challenging to describe how they learn and make decisions; hence, transparency and easy predictability are essential.

There is a growing corpus of research in the medical field that focusses on automated diagnosis and prognosis utilising machine learning [154]. Issues of inter-pretability are far more serious in the medical field than mere intellectual curiosity. Decisions involving human life necessitate a heightened level of attention to detail in areas where such matters are ordinarily disregarded. There is a lot at risk. Decision assistance for delirium, a very relative syndrome in the elderly, is being provided by AI forecasts that are both data-driven and context-sensitive. Using artificial intelligence algorithms to sift through EHRs in search of delirious patients is one such example. To determine an individual’s potential for delirium, such algorithms can factor in a plethora of patient-specific data, such as demographics, lab results, and drug usage [155]. User acceptance is still an open subject, despite the fact that these algorithms are highly effective on the data used for training set utilising [156]. Hence, it was necessary to incorporate an explanatory component that could adequately justify its predictions. The medical record of the individual provided crucial support, which helped build confidence and enhance patient safety. It follows that healthcare plays a crucial role in building trust and enhancing the security of patients [157].

### 13.1. The Necessity of Regulation

It has been demonstrated that deep neural network (DNNs) are delicate and susceptible to adversarial perturbation, even though they perform exceptionally well in result prediction. These perturbations led to the development of self-learned patterns in the Local Accessible ModelAgnostic explanation (LIME) model, which generated erroneous results. With respect to the input data, it generates synthetic data, trains a simple machine learning model that functions similarly to the intricate black-box model, and uses the model’s weights to assess the importance of features [158]. It has been demonstrated that DNNs are delicate and susceptible to adversarial perturbation, even though they perform exceptionally well in result prediction. Subsequent to being exposed to such disturbances, the Local Interpretable ModelAgnostic explanation (LIME) model learnt patterns that led to inaccurate outcomes. Utilising the input data, it generates synthetic data, builds a simple machine learning model (using the synthetic data) which functions similarly to the intricate black-box model, and uses the weight of this model to assess the importance of features [159].

### 13.2. The Necessity of Innovation

The explanation meets the continuous requirement to create increasingly highly sophisticated neural networks and effective algorithms. Researchers have just recently recognised that these algorithms are capable of learning new things. This new knowledge can be used to a number of fields, such as neurology and astronomy, to reveal hidden laws and new directions for research. Medical research has also verified predictions and preliminary results given by the LIME model along with other DNNs, even though they were not yet practical for people. Physicians can now recognise diseases and understand their patients’ conditions thanks to medical imaging technology. By exposing the fundamental layers of some black-box models that make up neural networks, XAI helps people and machines interact on a deeper level [160].

### 13.3. The Necessity of Advancement

Currently, technological developments like artificial intelligence (AI) are focused on becoming accepted and perfect. This demonstrates that the ongoing advancements in AI are essential to its success. Employing XAI, engineers will be guided to improve particular parameters by new understanding and insights into the system. One such instance is the implementation of the CrystalCandle tool in a sales team for Linkedin software which resulted in an 8% increase in subscription income [161].

## 14. Countering Adversarial Attacks and AI Evasion in Cybersecurity

The ability to identify and lessen risks has been greatly improved by the application of artificial intelligence (AI) into cybersecurity. Deep learning techniques and other machine learning models are frequently used for tasks like intrusions into networks prevention and virus identification. Adversarial attacks, which are malevolent attempts to take advantage of model flaws by introducing minute perturbations in input data that may result in misclassifications, have increasingly targeted AI systems themselves. The dependability and security of systems powered by AI are thus seriously threatened. Adversarial and AI-evasion assaults alter data in subtle, frequently imperceptible ways to trick AI algorithms. In security-critical settings, where even one incorrect categorisation could cause major damage, this can have dire repercussions. More robust defences are needed since these attacks are becoming more frequent and sophisticated [162].

Attacks by adversaries in machine learning Creating inputs that lead machine learning algorithms to produce inaccurate predictions is known as an adversarial attack. These attacks fall into two general categories: white-box as well as black-box techniques.

### 14.1. Adversarial Attack

1. White-box attacks: In this case, the model’s design, variables, and educating data are all fully accessible to the attacker. Because the attacker can provide extremely specific adversarial instances intended to fool the model, the white-box attacks are frequently more effective. This group of subjects includes methods like Projected Gradient Descent (PGD) and Fast Gradient Sign Method (FGSM).

2. Black-box attacks: In these, the attacker is unaware of the internal workings of the model. Rather, they create adversarial cases by querying the model and analysing its outputs. The transferability belongings, which allows adversarial instances created for an individual model to fool another irrespective of how the models’ architectures and training sets differ, is the foundation of black-box attacks. Some examples of black-box assaults are query-associated gradient estimation [163].

### 14.2. Current Mitigation Techniques

To improve model resilience, adversarial instances are incorporated into the training process. This method uses both regular data and adversarial instances to train the model. This aids in the model’s acquisition of accurate adversarial input classification. On the other hand, adversarial training might decrease performance on clean inputs and requires a lot of resources. Reducing the sensitivity of the model to input perturbations is known as defensive distillation [164]. Preprocessing inputs to eliminate potentially harmful disturbances is known as input sanitisation. Feature squeezing and au-toencoders are examples of input sanitisation algorithms that try to remove adversarial perturbations from inputs prior to the model processing them. These techniques may not completely stop sophisticated attacks, but they are helpful in lessening the effect of hostile noise. Gradient Masking: This technique reduces the effectiveness of gradient-based assaults such as FGSM and PGD by hiding or “masking” the model’s gradients. Gradient masking can not, however, offer total security because more advanced techniques, such as black-box assaults, can get around it. Anomaly Detection: To find potentially hostile inputs, anomaly detection techniques keep an eye on the input data and model outputs [165].

## 15. Innovational Models That Cater to the Needs of Individuals

Future models should be situated in real-world environments to Engage in physical connection with the environment to learn about human causal linkages. Some academics are interested in embodied AI, which refers to AI agents that can move and interact with their surroundings in three-dimensional (3D) virtual world simulations. Because they are always obtaining new observations from their surroundings that might aid in behaviour correction, embodied agents can learn in novel ways thanks to their interactivity. However, these agents cannot currently carry out routine duties like travelling around complicated settings, manipulating objects, or operating on patients due to the lack of maturity and robustness in current technologies. Furthermore, they are not yet secure enough to engage with people and the surroundings. Robots are anticipated to function autonomously and intelligently in the actual world in the future, accomplishing their objectives in a secure and dependable manner with the help of large-scale AIs. This could result in the creation of intelligent robot physicians who can use flexible limbs with multisensors to do extensive body exams and procedures, diagnose patients, and make clinical choices. However, issues like guaranteeing patient safety and the dependability of the robot’s decision-making processes remain to be resolved. It is also necessary to take into account ethical factors, such as the possibility of job loss for human healthcare practitioners. There are already indications that large language models can help with scientific research, and in the next months, we anticipate seeing more and more articles discussing ChatGPT’s application in this area.

ChatGPT’s remarkable capabilities and ease of use made it popular worldwide and enabled it to reach a significant milestone, elevating AI conversational instruments to a new level [166].

ChatGPT’s ability to comply with user instructions is a double-edged sword: on the one hand, this strategy makes it outstanding at communicating with humans, yet on the contrary, becoming subservient by origin reveals it to infringement, such as by producing persuasive resembling misinformation. Potential threats surfaced shortly after ChatGPT’s release.

This innovative technique may present potential as well as hazards for the medical research area.

The hazards associated with the fraudulent but unethical utilisation of LLMs in a medical context cannot be disregarded and should be evaluated proactively, as the field of science has not yet established the guidelines for a beneficial and secure use of this innovative technology.

## 16. Conclusions

In recent years, there has been a significant surge in attention regarding the applications of AI technology in critical pharmacy domains such as drug development, dosage design, polypharmacology, pharmacy services in hospitals, and more. This is due to the belief that AI techniques possess the ability to envision knowledge, solve problems, and make decisions, much like human beings. It has been demonstrated that automated processes and databases can be utilised to conduct effective analyses utilising AI techniques. The implementation of AI methodologies enables the development of novel hypotheses, techniques, predictions, and analyses of diverse related parameters to be accomplished effortlessly, affordably, and with reduced time investment. Pharmaceutical delivery technologies are undergoing a paradigm shift due to the influence of AI, which enables adaptive, personalised, and customised treatments. Through the utilisation of AI’s functionalities in analysing data, recognition of patterns, and optimisation, medical professionals and researchers in pharmaceuticals have the ability to optimise drug efficacy, mitigate adverse effects, and improve outcomes for patients. Pharmaceutical pharmacodynamics have been truly transformed by AI-based techniques. They provide numerous benefits in comparison to conventional experimental techniques. Pharmacokinetic variables can be predicted, drug distribution as well as clearance within the body can be simulated, and drug dosage and administration routes can be optimised using AI-based models.Although this prospective synopsis outlines promising prospects, it is critical to acknowledge that data quality concerns, laws and regulations, and moral standards must be resolved prior to the complete actualization of artificial intelligence’s capabilities in pharma manufacturing. Nevertheless, consistent progress and partnerships among scholarly institutions, sector, and regulatory agencies may enable AI-powered innovations to ultimately transform the pharmaceutical sector and enhance patient results in the coming years. In the context of general, the incorporation of AI technologies into the pharmaceutical sector has the potential to significantly advance the development of drugs, enhance patient outcomes, and bring about a paradigm shift, thereby facilitating the industry’s transition from era 4.0-era 5.0.

## Figures and Tables

**Table 1:** List of the ten most popular artificial intelligence models utilised by pharmacies.

Sr No.	Softwires Powered by AI	Application	References
1.	GAN (Generative Adversarial Networks)	1. Drug research and innovation makes extensive use of GANs for the generation of new chemical compounds and the optimisation of their attributes. 2. To generate structurally varied as well as functionally optimised potential drugs. 3. GANs use a generator network to produce new compounds and an evaluation network to assess its quality.	[9]
2.	RNN (Recurrent Neural Networks)	1. In the field of pharmaceutical research, RNN is frequently used for pattern-based activities like protein coding, dna data analysis, and protein configuration estimation. 2. It is able to learn features and then implementation into new patterns, capturing sequential relationships in the process.	[10]
3.	CNN (Convolutional Neural Networks)	1. Molecular structural analysis and the identification of possible therapeutic targets are two examples of based on images activities, where CNNs excel. 2. To help with medication development and receptor recognition, they may derive useful information through molecular pictures.	[11]
4.	LSTM (Long Short-Term Memory Networks)	1. When it comes to modelling and forecasting temporal relationships, LSTMs, a form of RNN, particularly emerge. 2. ADME Studies as well as pharmacodynamics of any drug have made quite easy to make drug profile by the use of this software.	[12]
5.	Graph Neural Networks (GNNs)	1. Drug discovery activities like the structure of molecules are well-suited to GNNs. 2. Their capabilities include molecular graph modelling, property prediction, simulated testing, and completely novel pharmaceutical development assistance.	[13]
6.	RL (Reinforcement Learning)	1. Drug dosage procedures and individualised treatment programmes have both benefited from the application of RL approaches. 2. RL techniques enhance medical results by learning from environmental interactions and making sequential judgements which assist in optimisation of dose.	[14]
7.	DQN (Deep Q-Networks)	1. By optimising drug development procedures through chemical activity prediction, DQNs—a hybrid of profound neural networks and reinforcement learning—have been employed. 2. Recommending promising subjects for additional testing.	[15,16]
8.	Bayesian Models	1. In order to quantify uncertainty and make decisions, the pharmaceutical industry uses Bayesian models like Gaussian processes and Bayesian networks. 2. Scientists can use them to optimise designs for experiments, conduct risk assessments, and generate probabilistic guesses.	[17,18]
9.	Autoencoders	1. The method for developing drugs makes use of autoencoders, which are autonomous neural models, to reduce multiplicity and retrieve features. 2. In addition to aiding in simulated analysis and combinatorial screening, they are able to capture crucial molecular properties.	[19,20]
10.	Transformer Models	1. One area where transformer models have found use is in the field of pharmaceutical natural language processing. One such model is BERT, which stands for Bidirectional Encoder Representations from Transformers. 2. Researchers can make better decisions about medication development with the help of their ability to retrieve useful information through data from clinical trials, data for patent, and also literature.	[21,22]

**Table 2:** AI tools used in drug discovery (all accessed on 20 January 2025).

Drug Discovery AI Tools	Information	Respective Websites
Chemputer	Improved structure for documenting the synthesis of chemicals	https://zenodo.org/record/1481731
ODDT	For application in the fields of molecular modelling & chemo informatics	https://github.com/oddt/oddt
ORGANIC	Molecules with certain properties can be synthesised using this instrument.	https://github.com/aspuru-guzik-group/ORGANIC
DeepChem	An artificial intelligence instrument to make drug discovery estimations that is built on Python	https://github.com/deepchem/deepchem
DeepNeuralNet-QSAR	Hypotheses concerning the action of molecules	https://github.com/Merck/DeepNeuralNet-QSAR
Neural Graph Fingerprints	Prediction for properties of newly discovered compounds	https://github.com/HIPS/neural-fingerprint
Hit Dexter	Predicting which compounds may react to biological experiments using artificial intelligence algorithms	http://hitdexter2.zbh.uni-hamburg.de
DeepTox	Assessment of the potential for toxicity along with biological compatibility	www.bioinf.jku.at/research/DeepTox
PotentialNet	An artificial graph convolutional artificial intelligent model for ligand binding.	https://pubs.acs.org/doi/full/10.1021/acscentsci.8b00507
REINVENT	Regenerative neural network-based molecular design	https://github.com/MarcusOlivecrona/REINVENT
DeltaVina	A scoring function for rescoring protein–ligand binding affinity	https://github.com/chengwang88/deltavina
AlphaFold	Forecasting the three-dimensional structure of proteins	https://deepmind.com/blog/alphafold

**Table 3:** Types of imaging used for medical purposes.

S.N	Imaging Type for Medical Purposes	Detailed Description
1.	X-rays (radiographic imaging)	Uses X-rays and other forms of ionising electromagnetic energy to produce images of things.
2.	Infrared imaging	Builds low-quality moving projection images of the physical internal structures in real time by repeatedly exposing them to X-rays at a reduced dose rate.
3.	Coronary angiography	Applied for the diagnosis of aortic aneurysms, stenosis, blockages, new vascular formation, and stent and catheter implantation.
4.	DEXA	The osteoporosis test uses Dual X-ray Absorptiometry, which is also called bone densitometry.
5.	CT Scan (Computed tomography)	Uses a computer and a lot of ionising radiation to create pictures of both solid and delicate tissues.
6.	MRI (Magnetic resonance imaging)	Imaging of the body made possible by a powerful magnet and radiofrequency waves; used in medical diagnostics.
7.	Ultrasound imaging	Makes use of broadband, sound waves with high frequencies reflected off of tissues to generate three-dimensional pictures.
8.	Bone scan	A method of monitoring bone repair that makes use of a radioactive substance.
9.	Electron microscopy	A high-resolution microscope that can magnify minute details.
10.	Radiation therapy (Nuclear Medicine)	Includes the use of nuclear characteristics for both medical evaluation and therapy.
11.	Magnetic resonance angiography scans	Draws blood vessel structures in the body with remarkable clarity.

**Table 4:** Computer programs used for patients’ data.

Computer Programmes (Software)	Target	Benefits	Drawbacks	PK/PD/Both	Reference
WinBUGS/Bayesian	In order to deal with information that is not yet quantifiable	Previous research can be utilised for model-fitting purposes without any additional processing.	Excessive processing time—Impossible negative results in specific PK/PD models	Both	[140]
Bayesian + PKBUGS + WinBUGS + version 1.3	Investigation of sirolimus concentration data using pharmacokinetic models	Possible covariate association identification; Simple integration of historical data with present-day data	Datasets are few and often lack useful information.	PK	[141]
Least Squares Support Vector Machine	Analysis of drug concentrations using patient profiles	A unique model is created for each individual patient—When compared to the PK modelling method, SVM-based approaches provide more accurate predictions of drug concentrations	Sample outliers significantly impact the model, reducing its accuracy.	PK	[142]
Random Sample Consensus (RANSAC) and Support Vector Machine with Drug Administration Decision Support System (DADSS)	Concentration, optimal dose, and dose interval prediction	Improved adaptability and structural adjustability; algorithmic predictability affected by dataset noise	Algorithm predictability is affected by dataset noise.	PK	[143]
Model Dependent Support Vector Machine/Profile SVM	Therapeutic medication tracking in patients undergoing kidney transplantation	an economical and crucial dosage Benefit nonlinear models	Extensive datasets—Time-consuming	PK	[144]
An SVM combined with a random forest model	How drugs interact with one another pharmacodynamically according to SES, CS, and TPC (Target Protein Connectedness)	The accuracy of the PDI predictions was 89.93% and the AUC value was 79.96%.	More extensive data processing and filtering is necessary.	PD	[145]
Gradient Boosting Machines, Random Forest, Linear Regressions (LASSO), and XGBoost	Forecasting the area under the curve (AUC) and plasma concentration-time series (0–24 h following multiple doses of rifampicin	Analyses that save time Covariate selections are made easier with this strategy.	Possible results that are not applicable to clinical practice	PK	[146]
XGBoost	Estimation of drug AUC of tacrolimus or mycophenolate mofetil (MMF)	PK datasets from renal, liver, and heart transplant patients were accurately predicted	Not possible to calculate the probability of target attainment and accurate dosing	PK	[147,148]
Simulated Annealing k-Nearest-Neighbor (SA-kNN)/Partial Least-Square (PLS)/Multiple Linear Regression (MLR)/Sybyl version 6.7	Prediction of pharmacokinetic parameters of antimicrobial agents based on molecular structure	Cost-effective—Requires less sample size	Requires multiple model generation methods—Interpretation of individual descriptors is almost impossible	Both	[149]
Drug Target Interaction Convolutional Neural Network (DTICNN)	Identification of drug-target interactions and prediction of potential drug molecules	Cost-effective—Time-saving	Large datasets are required	PD	[150]
Deep Long Short-Term Memory (DeepLSTM)	Computational methods to validate drug-target interactions	Based on Position Specific Scoring Matrix (PSSM) and Legendre Moment (LM)	Large datasets are required	PD	[151]

## Data Availability

No new data were generated or analyzed in this study. Data sharing is not applicable to this article.

## Abbreviations

AI	Artificial Intelligence
FDA	Food and Drug Administration
GAN	Generative Adversarial Networks
RNN	Recurrent Neural Networks
CNN	Convolutional Neural Networks
LSTM	Long Short-Term Memory Networks
GNN	Graph Neural Networks
RL	Reinforcement Learning
DQN	Deep Q-Networks
SAR	Structure-Activity Relationship
TF	Target Fishing Technology
ANN	Artificial Neural Networks
FES	Fuzzy Expert Systems
CT	Computed Tomography
AMM	Anonymized Mobility Maps
VDPV	Vaccine-Derived Poliovirus
SVM	Support Vector Machine
MPNN	Multilayer Perceptron Neural Network Classifier
CMC	Composite Monte Carlo
CF	Corrective Feedback
PNN	Polynomial Neural Network
DNN	Deep Neural Networks
